# Effect of a Standardised Dietary Restriction Protocol on Multiple Laboratory Strains of *Drosophila melanogaster*

**DOI:** 10.1371/journal.pone.0004067

**Published:** 2009-01-01

**Authors:** Richard C. Grandison, Richard Wong, Timothy M. Bass, Linda Partridge, Matthew D. W. Piper

**Affiliations:** UCL Institute of Healthy Ageing, Department of Genetics, Evolution and Environment, University College London, London, United Kingdom; Max-Planck-Institut fuer Neurobiologie, Germany

## Abstract

**Background:**

Outcomes of lifespan studies in model organisms are particularly susceptible to variations in technical procedures. This is especially true of dietary restriction, which is implemented in many different ways among laboratories.

**Principal Findings:**

In this study, we have examined the effect of laboratory stock maintenance, genotype differences and microbial infection on the ability of dietary restriction (DR) to extend life in the fruit fly *Drosophila melanogaster*. None of these factors block the DR effect.

**Conclusions:**

These data lend support to the idea that nutrient restriction genuinely extends lifespan in flies, and that any mechanistic discoveries made with this model are of potential relevance to the determinants of lifespan in other organisms.

## Introduction

In order to maximise its genetic contribution to posterity, an organism must appropriately direct the use of nutrients to traits such as growth, reproduction and repair. In some circumstances, this will mean maximising one trait at the expense of another. This idea has been used to explain the observation that relatively low food intake can result in longer life, because it comes at the cost of reduced rates of reproduction [1]–[4]. This particular trade-off phenomenon is widespread and has been termed dietary restriction or DR. Although extensively studied since its first description in 1935 [5], very little is known about the molecular details of exactly what resources are shared in this trade-off and how they are balanced between the traits. Uncovering these mechanisms has now become the holy grail of research into DR, with the aim of harnessing their power for longer and healthier lives.

One of the promising advances towards the goal of uncovering the mechanisms by which DR extends life was the discovery that the effect is evolutionarily conserved [6]–[10]. However, even with the use of short-lived model organisms for relatively rapid lifespan experiments, the mechanisms remain elusive. This is likely to be largely due to the complexity of physiology involved in determining length of life, but may be also in part due to technical issues in experimental design hampering a clear path of progress [11]. The ease with which complexity can be introduced into these studies can be illustrated by the large effects on fly lifespan caused by very small changes in nutrition. For example, substituting one source of the dietary yeast *Saccharomyces cerevisiae*, with another from a different supplier in an otherwise identical diet can have large effects on fly lifespan [12]. Similarly, lifespan differences have been reported due to the use of different bacterial strains as food for *Caenorhabditis elegans* [13] or by interchanging casein and soy peptone as the source of dietary protein for rodents [14]. In fact, a recent article has proposed that DR itself may have arisen as a by-product of laboratory life as animals are unintentionally subjected to selective breeding in the presence of an artificially rich nutritional environment [15]. Clearly, these issues need to be addressed if we are to uncover the molecular mechanisms of DR.

In our studies on DR in *Drosophila*, we have taken a systematic approach to optimise dietary composition such that fecundity and lifespan are maximised and any non-specific adverse effects of the food are avoided [12]. In this article, we extend this work to examine the effect of different techniques of long-term stock maintenance and microbial infection on the responses of ‘wild-type’ laboratory-maintained flies to DR. We have undertaken these experiments in order to establish a working protocol that avoids laboratory artefacts and will therefore aid studies seeking the molecular mechanisms of DR. As a result of performing these experiments with flies of different genetic backgrounds, we find interesting differences in the interaction between diet and genotype that form a solid basis for future work to uncover how DR extends the lifespan of flies and other organisms.

## Results

### An intermittent feeding regime did not affect Drosophila lifespan

We have previously published a description of the optimisation of a sugar/yeast (SY) medium for DR studies in flies [12]. This study found that yeast dilution in an otherwise unchanged medium effectively limits the flies' nutrient intake, decreases their daily and lifetime fecundity and increases their lifespan.

An alternative DR protocol that extends rodent lifespan is every other day feeding (EOD) [16], [17]. In these experiments, the EOD cohort has alternating bouts of 24 h access to unlimited food followed by 24 h starvation, while controls have continuous access to unlimited food. Interestingly, this intervention extends lifespan even though the EOD animals nearly fully compensated for the periods of starvation by eating more. Thus, intermittent periods of starvation could be equally as important as reduced nutrient intake for extending lifespan.

**Figure 1:**
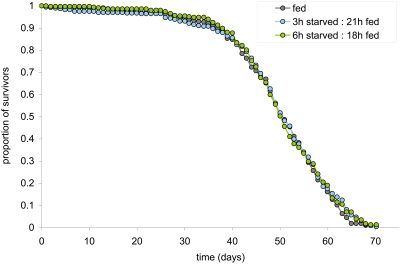
Intermittent exposure of flies to food does not increase their lifespan. Throughout adult life, Dahomey females were exposed to daily cycles of starvation∶feeding of either 3 h∶21 h or 6 h∶18 h. Neither treatment had any effect on lifespan. During the periods of starvation, flies had access to water only.

Two *Drosophila* studies have attempted a similar regimen and one reported a generally positive effect on lifespan when flies were subjected to 18 h access to food and 6 h access to water only in every 24 h [18]. In contrast, a more recent study has reported no positive effects of this treatment, or of any other treatments in which the timing of the starvation/feeding periods was altered [19]. However, in this latter study, the treatment was only implemented on 5 out of every 7 days of adult life, making it possible that any beneficial effects of the protocol were masked by the days without treatment. We therefore decided to test this technique using our laboratory strain Dahomey, applying daily bouts of either 3 h or 6 h starvation, during which the flies had access to water only. We found that neither treatment had a positive or negative effect on lifespan (Figure 1). While this could be taken to mean that DR does not work in flies, the lack of any effect on lifespan of the more severe restriction makes it impossible to know to what extent the flies were nutrient restricted or whether the periods of starvation were close to adequate to elicit a protective effect. Without a more extensive set of starvation periods, it is not possible to draw definitive conclusions about the effectiveness of this intervention in *Drosophila*.

### Comparison of the DR response between different laboratory strains

**Figure 2:**
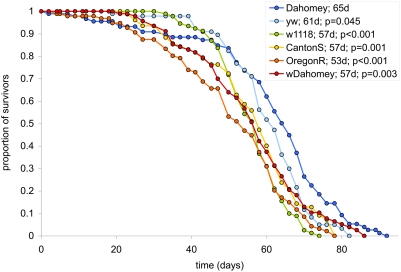
Different laboratory strains of wild-type Drosophila have different lifespans. Each genotype was raised in parallel under the same conditions and assayed on 1× SY for lifespan. All strains that were tested exhibited a shorter lifespan than our outbred laboratory strain Dahomey. The graph legend reports the strain name; median lifespan in days and; p-value from the log-rank test when compared to Dahomey.

In all of our DR optimisation experiments we have used our outbred laboratory strain of *Drosophila*, Dahomey. This strain has been maintained for many years on an SY diet in large population cages with overlapping generations. In contrast, most laboratory wild-type strains are largely inbred and maintained in relatively small numbers in individual containers and may have a varied nutritional history. Some of these housing conditions can easily lead to selection for early reproduction, which is known to cause shortened lifespan [20]–[22]. We therefore assayed the lifespan of several commonly used wild-type *Drosophila* strains on our standard SY food (1×; Figure 2). In all cases, the lifespans were significantly shorter than that of Dahomey and exhibited median lifespans from 53 days for OregonR to 65 days for Dahomey.

**Figure 3:**
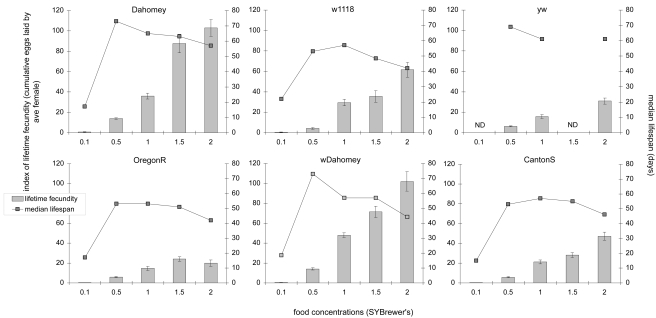
Different laboratory strains subject to DR. When tested in parallel under the same conditions, all wild-type strains tested exhibited a DR response. This is defined as a simultaneous increase in lifespan and decrease in lifetime fecundity when nutrient availability was reduced. Bars: index of lifetime fecundity±standard error of the mean; connected points: median lifespan in days; ND: not determined. Data shown are from a single trial in which all lifespans were run simultaneously. They are representative of triplicate data sets for Dahomey, CantonS and OregonR and duplicates for yw; w118 and wDahomey data are from a single trial.

Next we asked what the effect of this variation was on the DR response in these different strains. This was both to assess how our DR protocol is likely to behave when implemented in other laboratories that routinely use fly stocks other than Dahomey, as well as to look for strains with altered DR responses that might provide insights into its mode of action. The operational definition of DR is the range of nutrition that causes lifespan to increase and fecundity to decrease [23]. It should be noted that this definition excludes the dilution from 0.5× down to 0.1×, as this caused the flies to become malnourished and both lifespan and fecundity to decrease (Figure 3). For Dahomey and wDahomey, the DR range was from 2× to 0.5× food, while for w1118 and CantonS it was from 2× to 1×, and for OregonR was from 1.5× to 0.5× (Figure 3). For OregonR only, the highest food concentration caused egg laying to decrease, which indicated that the associated lifespan decrease from 1.5× to 2× was not accompanied by increased intake of biologically valuable nutrition and therefore could be due to a non-specific detrimental effect of high food. It was thus considered outside of the functional DR range for this strain. Finally, for yw, there was a clear DR response from 1× to 0.5× food but, owing to incomplete data, we cannot report any possible broader DR effect. Thus in all cases, a DR response was observed under these conditions although its exact nature was different for different wild-type strains.

In all comparisons from all trials, Dahomey, wDahomey and yw exhibited the longest lifespan (Table 1) with medians from 69 to 73 days over different trials on 0.5× food (Dahomey v wDahomey, p = 0.69; Dahomey or wDahomey v highest median lifespan from each other genotype, p<0.001, log-rank test). Dahomey and wDahomey also exhibited higher reproductive output than the other wild-types at each food concentrations except 0.1×, as well as the maximum reproductive output from all conditions (on 2× food) (Dahomey v wDahomey, p = 0.97; Dahomey or wDahomey v highest reproductive output for each other genotype, p<0.003, Wilcoxon rank-sum test).

**Table 1:** 

Wild-type strain	Food Conc (x)	Median lifespans^1^			ave lifespan change due to DR^2^
		Trial 1	Trial 2	Trial 3	
Dahomey	0.1	17.1	18	ND	
	0.5	**73**	**73.5**	**69.1**	
	1	65	66.5	59.5	31%
	1.5	63	64	48	
	2	*57*	*55*	*52.5*	
yw	0.1	ND	ND	ND	
	0.5	**69**	ND	**73.5**	
	1	*61*	ND	*66.5*	12%
	1.5	ND	ND	ND	
	2	61	ND	48	
w1118	0.1	22	ND	ND	
	0.5	53.1	ND	ND	
	1	**57**	ND	ND	36%
	1.5	48.4	ND	ND	
	2	*42*	ND	ND	
CantonS	0.1	15	18	ND	
	0.5	53.1	**57** 3	**50**	
	1	**57**	**59.5**	48	28%
	1.5	55.1	52.5	*38.5*	
	2	*46.1*	*45.5*	*38.5*	
OregonR	0.1	17	22	ND	
	0.5	**53.1**	45.5	**66.5**	
	1	**53.1**	**52.5**	59.5	15%
	1.5	*50.9*	*48*	*55*	
	2	42	45.5	52.5	
wDahomey	0.1	18.5	ND	ND	
	0.5	**73**	ND	ND	
	1	57	ND	ND	66%
	1.5	57	ND	ND	
	2	*44.1*	ND	ND	

1 bold numbers denote the greatest median lifespans and italicised numbers the shortest median lifespans, within the DR food range for that strain in that trial.

2 For all DR ranges for each strain, the longest-lived condition was significantly different from the shortest-lived condition; percentages are derived from the average lifespan difference due to DR.

3 In cases where there was no significant difference between two food types for the longest or shortest-lived condition, two numbers are in bold or italicised.

### Effect of tetracycline treatment on the DR effect

*Drosophila* are host to a range of microbes, and for many strains, this includes a bacterium of the genus *Wolbachia* that resides in the cytoplasm of reproductive tissues [24]. In some cases, the presence of *Wolbachia* has been shown to alter lifespan [25]. Recently, a vertically inherited factor that was curable by tetracycline treatment was shown to account for at least part of the long lifespan of a long-lived *Drosophila* mutant [26]. We decided it was important to examine the effect of such infections on DR, because if they account for the lifespan difference, it is unlikely DR in *Drosophila* is useful as a model for higher organisms.

**Figure 4:**
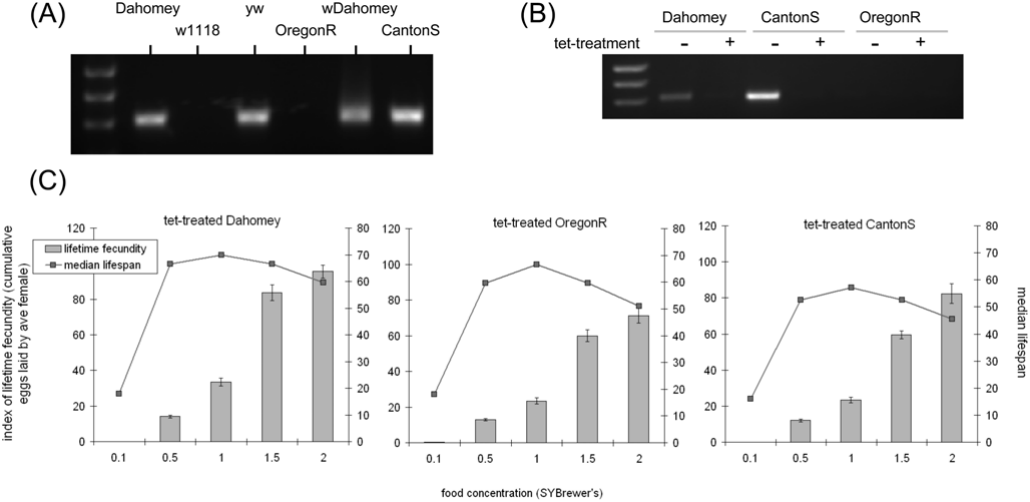
Tetracycline treatment does not eliminate the DR response. (A) Gel showing diagnostic PCR for the presence of the intracellular bacterium *Wolbachia*; (B) three strains were selected from the set of wild types for treatment with tetracycline, which was sufficient to clear *Wolbachia* if present. (C) Each of the three strains was then allowed at least five generations to recover on non-tetracycline-containing food before being assayed for lifespan and fecundity on different concentrations of food. Each of the three strains still exhibited a DR response after tetracycline treatment. Bars: index of lifetime fecundity±standard error of the mean; connected points: median lifespan in days. Data shown are from one of two trials in which all lifespans were run simultaneously.

Upon testing our wild-type strains for *Wolbachia* we found all except w1118 and OregonR were infected (Figure 4a). Therefore, because all strains exhibited a DR response, *Wolbachia* infection *per se* can not account for the full effect of nutrition on lifespan. To test if tetracycline-treatment could eliminate the DR response by other means, we selected three lines for treatment (Dahomey, CantonS and OregonR). After two generations on tetracycline-containing food, flies were subsequently maintained on normal food to recover for at least five generations. PCR testing revealed that the treatment was effective as both Dahomey and CantonS were cleared of *Wolbachia* (Figure 4b). When subjected to different food concentrations, all three tetracycline-treated lines retained their DR response (Figure 4c). In the trial shown, the lifespan peak for all three strains was at 1× food and fecundity increased to 2× food. While this was qualitatively different from that seen in the previous trials with non-tetracycline-treated flies, a further trial with these lines after an additional five generations on normal food, revealed more similar data to that shown in Figure 3 (data not shown). Thus, tetracycline-treatment may produce a transitory alteration in the way flies respond to food, but its effects can not account for the DR response.

## Discussion

### Intermittent feeding did not extend fly lifespan but does not rule out DR in flies

There are several different ways to restrict the access of animals to nutrition and thus extend lifespan by DR [27]. For flies, dilution of the concentration of yeast in a diet that is provided in excess, has proven to be practical and effective [12], [28]. However, food dilution methods are unique to the invertebrates and in mammalian studies, periodic access to food is used. One such protocol provides animals with a measured amount of food that is completely consumed before the next meal. While effective for extending rodent lifespan [7], it has been unsuccessful when used on flies [29], [30]. An alternative technique is EOD feeding, which extends rodent lifespan by alternating periods of access to excess food with periods of starvation. Importantly, the mice subjected to this regime increased their feeding behaviour such that they consumed nearly the same quantity of nutrients as controls. Thus, regular periods without food maybe just as important as reducing nutrient intake for extending rodent lifespan [16]. In contrast, this protocol has had little or no success when adapted for flies [18], [19]. In this study, we also found no extension of life using a similar protocol on *Drosophila* (Figure 1). Thus, our results support the previous invertebrate data and could be used to argue that periods of starvation cannot extend the lifespan of flies [30], [31] or that the mechanism by which DR extends lifespan is different between flies and mammals. While both of these explanations are possible, the fact that lifespan was not shortened by the more severe of our restriction treatments means we are unable to determine how much nutrient intake may have been reduced, or exactly what other periods of starvation could be protective for lifespan in our flies. While a more extensive range of starvation periods would be revealing, other factors such as the time of day at which food is removed may also be important since feeding behaviour is controlled by the circadian rhythm [32]. Thus, it is easy to implement an inappropriate methodology when attempting to DR flies in this way and the absence of a positive result does not rule out the possibility of observing a positive effect if protocols were optimised.

**Figure 5:**
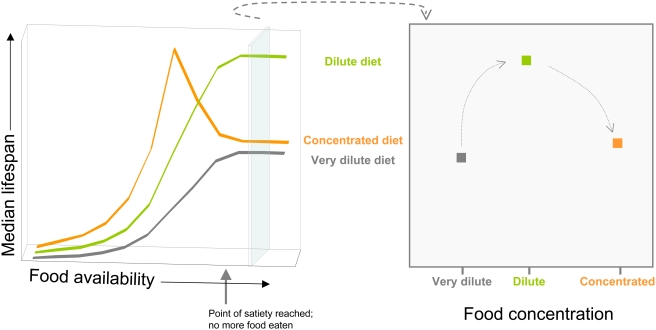
Model of the relationship between lifespan and DR protocols that reduce access to food either by intermittent exposure (left panel) or nutrient dilution (right panel). These demonstrate how the composition of food used for intermittent feeding protocols could lead to the false conclusion that DR does not exist for an organism. Three different diets are shown that vary in a given nutrient concentration from ‘very dilute’ to ‘concentrated’. In this example, increasing access to the concentrated diet causes lifespan to rise to a peak (DR) beyond which lifespan decreases. At some point (marked here as the ‘point of satiety’) the animal will no longer be able to eat any more food, meaning the nutrition level it experiences is capped and no further increase in availability will further decrease lifespan. For the dilute and very dilute diets, the point of satiety is reached before the level of nutrients ingested has a chance to cause lifespan to reduce. Thus, there is no lifespan increase for any intermediate level of food restriction, making it look like the organism does not exhibit a DR response. For flies, these problems can be avoided by assaying lifespan in the presence of excess food that is diluted to differing extents. The relationship of this situation to DR by intermittent feeding is represented by taking a cross-section through the graph on the left. The plot on the right shows the type of data presented herein and for other invertebrate studies.

If nutrient restriction is the critical factor in these DR experiments then intermittent feeding protocols that use different dietary compositions would also be expected to vary lifespan outcomes in different ways. Figure 5 illustrates how this is possible. When given increasing doses of a relatively concentrated diet (orange line), lifespan would increase as malnutrition lessens to a peak at an intermediate level of food availability. As food availability is increased beyond this point, lifespan decreases via the DR response. At some point, no additional increase in food availability will further shorten lifespan as the organism will reach its limit to ingest more food (‘point of satiety’ and beyond). If, however, the concentration of the food being provided is low enough (represented by the ‘dilute’ and ‘very dilute’ diets in Figure 5), lifespan will increase to a plateau whose onset occurs at the point that the organism's food intake limit is reached. If these dilute food types are used in an intermittent feeding protocol, it would be impossible to find an intermediate level of food exposure which increases lifespan, falsely giving the impression that DR does not exist. It is possible that this can explain why some studies have been published that did not find a DR response (eg [33]–[35]). As mentioned above, food dilution has proven to be the most successful intervention to implement DR in flies [11]. The connection between this intervention, where the food remains in excess, and intermittent feeding can be found by taking the lifespan values at any one level of food availability above the point of satiety in the left panel of Figure 5. A cross-section of these values is shown in the right panel of Figure 5; this represents the standard DR effect in flies (eg Figure 2). It should be noted that in reality, this illustration is somewhat simplistic in that the lifespan-sensitive nutrients represented on the x-axis are unlikely to be accurately represented by the term ‘food availability’. Furthermore, nutrient composition variations are likely to alter the point of onset of satiety, which in turn changes the onset of the lifespan plateau. Thus, although further work on diet composition, feeding intervals and measured food availability may uncover an alternative intermittent feeding regime suitable for flies, it is likely to be a labour intensive process that may not provide any more information about DR than dietary dilution.

### DR in Drosophila does not appear to be a laboratory artefact

**Figure 6:**
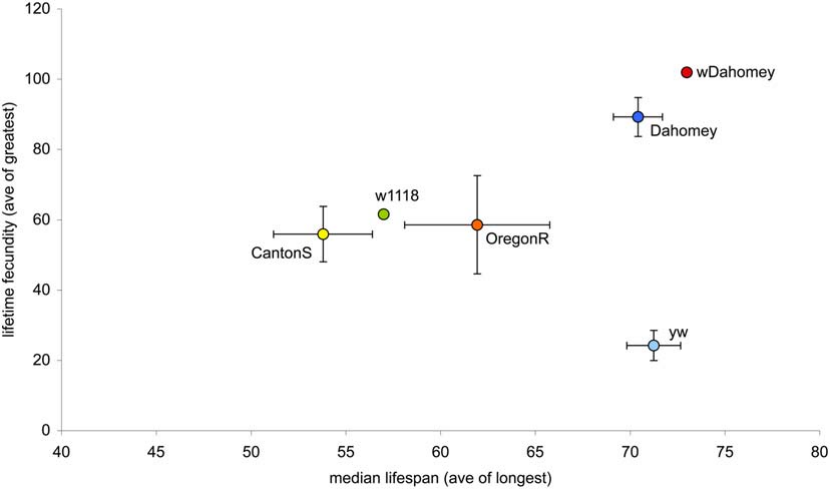
The Dahomey genetic background is capable of the longest lifespan and greatest reproductive output of the wild-type strains tested. For median lifespan, the data are the averages from the longest lived conditions for each strain. For lifetime fecundity they are the average of the condition producing the greatest lifetime reproduction. It should be noted that the conditions under which these occur is different for the two traits, as predicted by the expectations of DR, and that they may be different for each different strain. Data from n independent repeats, where n = 5 for Dahomey, CantonS and OregonR; n = 2 for yw, and; n = 1 for w1118 and wDahomey.

For ease of handling and to extend generation times, fly stocks in the laboratory are often kept in small numbers, under relatively poor nutrient conditions and at low temperatures. Over time, these factors are likely to exert selective pressures that could influence lifespan. Importantly, when transferring stocks to fresh food for maintenance, it is relatively easy to select for early age of reproduction, which is known to reduce adult lifespan [36]. That this happens in the laboratory has been demonstrated by comparing the lifespans of flies maintained for years in the laboratory under normal stock-handling conditions with others selected for early or late reproduction as well as others freshly caught from the wild [37]. This study showed that the laboratory stocks were as short lived as those selected for early reproduction, while the wild-caught lines had a much longer lifespan, similar to flies selected for late reproduction and were much longer lived. In our laboratory, we have maintained a wild-type outbred stock (Dahomey) since 1970 in large population cages with overlapping generations. When compared with other laboratory wild-type strains that we have maintained using routine stock handling techniques, we found that Dahomey demonstrated the capacity for both the longest lifespan and the greatest lifetime egg laying output (Figure 6). Thus, maintenance of flies using large population cages with overlapping generations appears to preserve the life history characteristics of wild-flies for long periods of time. This is in agreement with previous work that demonstrated this fact for flies maintained in the laboratory during a three year period [38].

Importantly, despite the differences between strains in their selection histories, all exhibited a DR response (Figure 3). Recently, it has been proposed from work with mice that lifespan extension by DR could simply be an artefact of laboratory domestication because a wild-caught strain was reported whose longevity was not increased in response to a typical DR regime [15]. In contrast, a recent study of several wild-derived strains of *C. elegans* showed that all exhibited a DR response [39]. Although we have not directly tested DR using flies recently caught from the wild, our study indicates that they would exhibit a DR response because of the strong effect seen with Dahomey (Figure 3 and Figure 4). It should be noted that the invertebrate studies were conducted using a DR technique that deprived worms of bacteria, while the rodent study used a food restriction protocol with only one level of limitation. Thus, as explained above, diet design and an incomplete range of food concentrations could be important factors in explaining why the DR effect was apparently absent from wild mice [15].

### DR in Drosophila is not sensitive to tetracycline treatment, but varies with diet quality and genotype

We show here that DR is not sensitive to infection with the bacterium *Wolbachia*, or indeed any other tetracycline-sensitive infection that may be present in flies (Figure 4). Interestingly, the levels of fecundity at a given food concentration differed after tetracycline treatment (compare Figure 3 with Figure 4c). This indicates that some tetracycline-sensitive microbes carried by some flies might be involved in the control of fecundity. However, further data would be required to validate this observation since a subsequent trial with the tetracycline-treated flies saw fecundity levels return to those previously observed (as for Figure 3; data not shown). Together, these data extend previous work we have performed to optimise a DR protocol to avoid lifespan variations from non-nutrient dependent effects [12]. From this work, we have sought to generate a standardised DR protocol to aid studies into the mechanisms of DR. However, we here report that the food concentration to yield the longest lifespan in Dahomey was at 0.5×, which is less than the 1× reported in [12]. This demonstrates an inherent problem with using a natural ingredient like yeast whose nutritional content varies seasonally due to production methodology and the quality of its feedstock. In doing so, it also highlights the need for a standardised synthetic defined medium to replace yeast-based diets to study the details of how lifespan varies with food composition. Interestingly, not all strains exhibited a lifespan peak at the same food concentration as Dahomey (Figure 3). It is already known that genotype can affect the interaction between lifespan and food [27], [40]–[42] and could indicate the breadth of the DR effect on fly health. One interesting possibility from these data is that if flies of different genotypes die from different pathologies, DR has the ability to delay the onset of each of these causes of death, which agrees with data from rodent studies [7], [43]. Future work on the exact molecular mechanisms of DR via interactions with different genotypes on precise dietary manipulations will be key to exploring this further.

## Materials and Methods

### Fly stocks and maintenance

Dahomey: This strain has been in the laboratory since 1970, having been collected in West Africa in what is now the Republic of Benin. Four population cages (dimensions: 20 cm H×21 cm W×30 cm D) have been maintained in parallel at 25°C on a 12-hour light/dark cycle. At all times, 12 bottles of food are in each cage, being replaced gradually. Each week, three half-pint bottles containing 70 ml of food (1× SY) are supplied to each cage and the three oldest bottles removed.

wDahomey was generated by backcrossing the white gene from w1118 into the Dahomey genetic background. It has since been maintained in one large population cage with a feeding regime as described above for Dahomey.

w1118, yw, OregonR and CantonS have been maintained in the lab for many years under a variety of conditions. Generally, this involves transferring each new generation to a fresh set of several half-pint bottles or vials of food. These are usually kept at 18°C to extend each generation's lifecycle and are fed either 1× SY food or a cornmeal-based diet (see below).

### Media

The SY food reported here is the same as SYBrewer's in [12]. Standard (1×) contains per litre: 100 g autolysed Brewer's Yeast (MP Biomedicals, Solon, OH), 100 g sucrose (Tate & Lyle sugars, London, UK), 15 g agar (Sigma, Dorset, UK), 3 ml propionic acid (Sigma, Dorset, UK), 30 ml Nipagin M solution (100 g/l methyl 4-hydroxybenzoate in 95% ethanol) (Clariant UK Ltd, Pontypridd, UK), distilled water to 1 l. Cornmeal-based diet used in stock keeping contains: 60 g cornmeal (organic polenta; B.T.P. Drewitt, London, UK), 20 g autolysed Brewer's yeast, 85 g sucrose, 10 g agar, 25 ml Nipagin M and 1 l distilled water.

Tetracycline treatment was carried out by the addition of 25 µg/ml tetracycline to 1× SY food for two generations.

For stock maintenance, food was cooked in a 60 l Joni Multimix food preparation kettle (Joni Foodline, Munkebo, Denmark), while experimental food was prepared on a gas hob as described in [12].

### Lifespan and fecundity assays

Flies were reared at a standard density for at least two generations before being used for lifespan experiments as previously described [12], [44]. All experiments were performed with female flies that were allowed 48 h to mate after emerging as adults. On the second day of adult life, flies were lightly anaesthetized with CO_2_, sorted and counted at 10 per vial. The minimum number of flies per condition was 100. For the intermittent feeding experiment, five replicate 1 l cages, each containing 100 flies was used for each condition. These cages have two side-arm inlets that can each accommodate a food vial. The periods of starvation were initiated at 10:00 (lights on), whereupon the food vial (2× SYBrewer's) was replaced with an empty vial. In all cages at all times, flies had constant access to a vial containing water that was plugged with wet cotton wool. This was housed in the side-arm not containing the food vial. In all cases, flies were transferred to fresh food at least three times a week, at which point deaths were scored.

For fecundity measurements, eggs were counted after the flies had been in the vials for between 18 and 24 h. Generally, these counts were performed once a week for the first six to seven weeks of adult life. Importantly, the first egg count was only conducted after at least four days exposure to the new food in order to allow time to adjust to the new nutritional conditions.

### PCR detection of Wolbachia infection

PCR for detection of Wolbachia was performed using primers wsp81F and wsp691R (kind gift from G. D. D. Hurst) as described in [45]. In each case, a sample of flies form the experimental generation was used for PCR testing.

### Data analyses

Lifespans were recorded and analysed using spreadsheets created in-house in Excel. Comparisons using the Wilcoxon rank sum test were performed in R, v2.5.1 [46].
